# Postoperative recurrence of desmoid tumors: clinical and pathological perspectives

**DOI:** 10.1186/s12957-015-0450-8

**Published:** 2015-02-07

**Authors:** Yi-fei Wang, Wei Guo, Kun-kun Sun, Rong-li Yang, Xiao-dong Tang, Tao Ji, Shun Tang

**Affiliations:** Musculoskeletal Tumor Center, Peking University People’s Hospital, Beijing, 100044 P.R. China; Department of Pathology, Peking University People’s Hospital, Beijing, 100044 P.R. China

**Keywords:** Histopathology, Immunohistochemistry, Tumor resection

## Abstract

**Background:**

The clinical features and the pathological changes of desmoid tumors were studied to point out the key factors affecting the recurrence.

**Methods:**

The clinical data and specimens of 56 patients who underwent desmoid tumor resection from 2003 to 2008 were reviewed. Possible clinical factors related to the postoperative recurrence were analyzed statistically. The specimens round the lesions were studied histopathologically.

**Results:**

The overall recurrence rate was 39.3%. The postoperative recurrence rate of the patients with negative surgical margins and no tumor invasion of the major vessels and nerves was low (*P* < 0.05). However, the desmoid tumors could destroy the cortical bone and invade the medullary cavity.

**Conclusions:**

Desmoid tumors were pathologically benign, which could extensively invade tissues around the lesions. The invasion of major vessels and nerves and quality of surgical margins are the key factors for the high postoperative recurrence rate.

## Background

Desmoid tumors, also known as hard fibroma, fibromatosis, or aggressive fibromatosis, are rare soft tissue tumors. Although desmoid tumors have few mitotic figures, their typical malignant features, such as a lack of distant metastatic potential, locally aggressive growth, and invasion of the surrounding tissues, make the full resection very hard. Besides, their recurrence rate was estimated ranging from 19% to 77% [1]. Surgery, in general, was the major treatment for desmoid tumors. Moreover, the combination of surgery, radiotherapy, chemotherapy, and endocrinal therapy also has the ability to improve the clinical outcomes [2]. The quality of the surgical margin was fatherly treated as the sole factor affecting the local control rate [3]. The residue usually locates between the tumor and the normal structures.

Noticeably, in order to increase the quality of the resection margins and decrease the recurrence rate, it is of importance to study the invasion mechanism of desmoid tumors into the surrounding structures. However, as far as we know, reports about the clinical features of desmoid tumors and the biological behaviors of the surroundings are sporadic. Thus, we collected the clinical data and pathological specimens of 56 desmoid tumor patients with complete postoperative follow-up treatment, analyzed the clinical features of the desmoid tumors, and studied histopathologically their surrounding structures to investigate the related clinical factors and pathological mechanisms causing the postoperative recurrence. Our aim was to provide the evidences for the surgical treatment strategies.

## Methods

### Clinical data

Clinical data and pathological specimens of 56 desmoid tumor patients were collected at the Musculoskeletal Tumor Center, Peking University People’s Hospital, P. R. China, from February 2003 to December 2008. All the patients returned for follow-up during a period ranging from 33 to 108 months ending with tumor recurrence. None of them was offered any preoperative radiotherapy or pharmacotherapy. Twenty-six patients with primary tumors and 30 with recurrent tumors were categorized as primary and recurrent group, respectively. Among them, 20 patients were male and 36 were female. The youngest patient was 7 years old and the oldest was 76 (median 29.3). Lesions located either in the trunk (16 cases) or in the limb (40 cases) (Table 1). The 16 trunk cases included eight pelvis, three retroperitoneals, three backs, and two necks, whereas the 40 cases of limb comprised 15 lower arms, 13 upper arms, eight hips, and four shoulders.

**Table 1:** **General data of the 56 patients with desmoid tumors**

**Categories**	**Number of patients with primary tumor**	**Number of patients with recurrent tumor**
Gender		
Male	8	12
Female	18	18
Patient age		
≥ 30	10	16
< 30	16	14
Tumor site		
Limb	16	24
Trunk	10	6

### Surgical treatment

All the surgeries were conducted by two senior surgeons at the Musculoskeletal Tumor Center, Peking University People’s Hospital, P. R. China. Tumors with pure soft tissue involvement would receive a gross total resection, whereas tumors invading major vessels and nerves, such as popliteal vessels, sciatic nerve, and brachial plexitis, would be preserved as much as possible after separation. If the tumors fully circumscribed, the surrounding structures need to be removed completely to achieve the satisfactory clinical margins. Artificial vascular graft would be deployed if needed. In our study, the tumor sites included eight sciatic nerves, four vascular nerve axillas, two popliteal vessels, three ulnar nerves, three iliac vessels, two neurovasculars, three median nerves, two nervus peroneus communis, two radial nerves, two tibial nerves, two vascular nerves, one lumbar nerve, one femoral nerve, and one carotid artery and vein, respectively. Lesion curettage would be offered if the tumors had bone involvement. However, if the tumors circumscribed and infected the diaphysis aggressively or the bone was severely destroyed, the tumor segmental resection, inactivation followed by bone graft and internal fixation, or prosthetic replacement would be conducted (Table 2). Patients with positive margins received radiotherapy at a dose of 50 Gy [4], whereas the others were administrated with NSAIDs postoperatively. NSAIDs included Celecoxib® (Pfizer, Inc., NY, USA) at a dose of 200 mg bid [5] and Raloxifene (Hongfuda Pharmaceutical Chemical Ltd., Shandong, P. R. China) at a dose of 200 mg qd. Noteworthy, Raloxifene is not eligible for pregnancy, pregnancy planning, or immaturity [6,7]. Chemotherapy alone was not applied as the treatment because it would probably make the surgery more difficult, put the patients at risk of eccyliosis and limb contracture, and even worse, induce tumor malignancy.

**Table 2:** **Cases and surgical treatment of desmoid tumors with bone involvement**

**Tumor sites**	**Cases**	**Surgical treatments**
Ilium and acetabulum	4	3 for lesion curettage, 1 for lesion curettage with bone grafting
Ulna and radius	3	2 for tumor segmental resection, inactivated with bone grafting and internal fixation, 1 for tumor segmental resection with radius replaced by fibula
Pubis	3	2 for lesion curettage, 1 for inactivated bone grafting with internal fixation
Humerus	3	2 for lesion curettage with internal fixation, 1 for tumor resection with prosthetic replacement
Femur	2	1 for tumor resection with prosthetic replacement, 1 for lesion curettage with internal fixation
Clavicle	1	Lesion curettage
Tibia	1	Lesion curettage with internal fixation

### Pathological examination

Cross sections of desmoid tumors were made horizontally and vertically, and then the tumors and their surroundings were later observed. Different sections and their around apparently normal tissue were sampled in the boundary between the tumor and the adipose tissue, muscle, anadesma, ligament, vessel, nerve, or bone. All specimens were fixed for 24 h in formalin solution. Besides, the specimens with bone involvement were decalcificated for at least another 48 h. Samples were sliced, 4 μm in width, after paraffin embedding. All the slices were confirmed histologically: the pathological morphology of desmoid tumors and their surroundings were observed under the microscope by hematoxylin-eosin staining (HE). The immunohistological staining method, EnVision® two steps (Beijing Zhongshan Jinqiao Biotechnology Ltd., China), was then applied following the manufacturer’s instruction. TBS was used as blank control, and the positive slice was used as the positive control according to the manufacturer’s recommendation. Positive outcomes were determined if brownish yellow to brown particles occurred in more than 10% of desmoid tumor cell nuclei.

### Postoperative follow-up

The patients with desmoid tumors were chosen to receive clinical follow-up during a postoperative period of the 3rd, 6th, 9th, 12th, 18th, and 24th month, respectively. Physical and radiological examination, such as MRI or CT, would be deployed once a year afterward. The postoperative recurrence was determined by the recurrent encapsulation of the surgical site, the radiological examination, and the pathological diagnosis. Tumor size was measured based on the preoperative MRI or CT images. The margin quality was categorized as negative margins (R0) and the macroscopic or microscopic residues of pathological changes (R1/2).

### Statistical analysis

All the clinical and surgical treatment, pathological examination, and postoperative follow-up were approved by the Ethics Committee at the Musculoskeletal Tumor Center, Peking University People’s Hospital, P. R. China (No.PUPH2012900401). Gender, patient age, primary treatment or recurrence, tumor site, tumor size, invasion of major vessels and nerves identified by preoperative examination or surgery, bone involvement, quality of surgical margins, adjuvant radiotherapy, and pharmacotherapy or not were analyzed statistically by SPSS 19.0. The *X*^2^ test was used for univariate analysis. The multi-factors logistic regression analysis would be deployed for the independent factor determination if *P* < 0.10 in univariate analysis. A *P* value of <0.05 was considered significant for all analyses.

## Results

### Pathological examination

**Figure 1:**
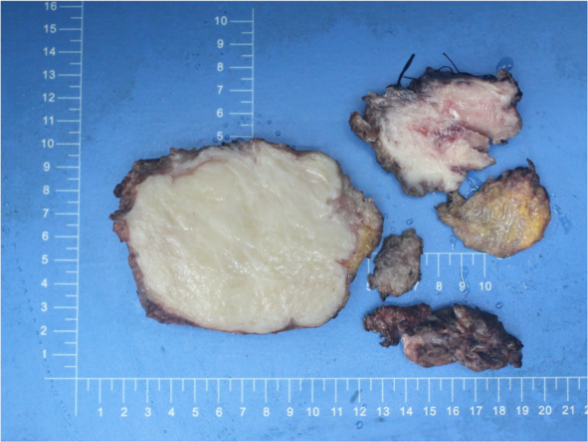
**Desmoid tumor in the proximal part of the left thigh of a 29-year-old woman.** Macroscopically, desmoid tumors were yellowish white on the cut and often poorly circumscribed.

In general, the pathological changes were different in size ranging from 3 to 17 cm (median 7 cm). Desmoid tumors with opaque margins invaded the surrounding muscles, adipose tissue, anadesma, ligaments, vessels, and nerves, where the connection was tight and hard to separate. The depression of bone surface pressed by the tumor located at the connecting site in between. Macroscopically, the tumors were tenacious, yellowish white on the cut. It was hard to distinguish them from the scar tissue and ligament (Figure 1).

### Histological characteristics

**Figure 2:**
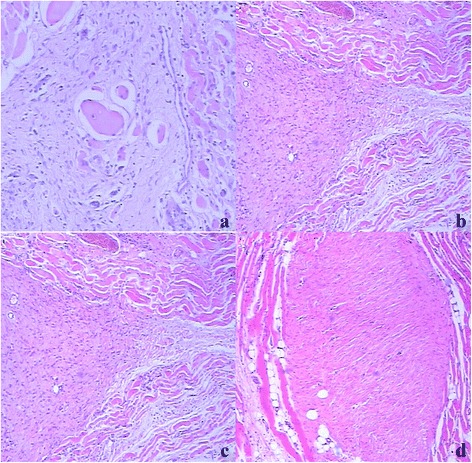
**Histological features of desmoid tumors in the proximal part of the left thigh of a 29-year-old woman (arrows) (a–d). (a)** Desmoid tumors invaded into the skeletal striated muscle aggressively. Degeneration of skeletal muscle cells could be seen (HE × 100). **(b)** Budding-like protrusion of the lesions invading into the muscles could be seen on the juncture of tumors and muscles (HE × 40). **(c)** Isolated small lesions in muscles were found away from the main part of the tumor (HE × 40). **(d)** Microscopically, desmoid tumors were poorly circumscribed on tumor-ligament boundary (HE × 40).

Microscopically, desmoid cells were spindle shaped or swollen to some extent caused by cell proliferation among massive collagen, small vessels, and the round edema fibrous connective tissue (Figure 2a, arrows). Opaque tumor cells had spindle to short spindle-shaped nuclei with abundant cytoplasm. These cells were slightly dysmorphic, arranging in bundles, accompanied by rare nuclei mitotic figures occasionally with mucoid degeneration and hyalinization (Figure 2a).

**Figure 3:**
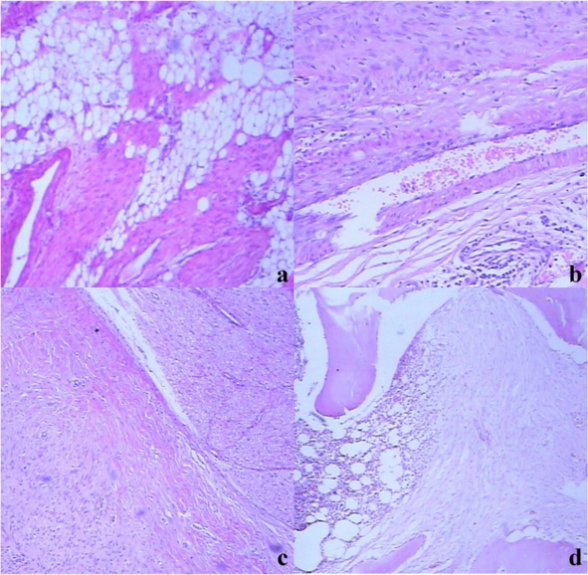
**Histological features of postoperative recurrent desmoid tumors in the right forearm of a 15-year-old man (arrows) (a–d). (a)** Lesions with adipose tissue involvement (HE × 40). **(b)** Desmoid tumors around vessels could not invade into the vessel wall to form tumor thrombus (HE × 40). **(c)** Desmoid tumors invaded into the connective tissue and perineurium around nerve tissue (HE × 40). **(d)** Desmoid tumors with bone involvement penetrated into the periosteum and cortical bone and invaded into the bone marrow cavity along the bone trabecula (HE × 40).

The muscle invasion and the degeneration of the muscle cells were found around the tumors. Consequently, the necrotic muscle cells were replaced by the tumor cells (Figure 2a). Budding-like protrusions of the lesions were visibly invading into the muscles along the connective tissues in the muscle bundles on the juncture of tumors and muscles (Figure 2b). Isolated small lesions in muscles were also found away from the main part of the tumors (Figure 2c). When the tumors invaded the anadesma, ligament, and scar tissue, there were no clear margins in between (Figure 2d). Nevertheless, it was even harder to separate the tumor cells from the normal muscle tendon cells based on the cell morphology. The tumor identification can only be made by the careful observation of the regular arrangement of the cells and the progression of the tumors. Lesions with adipose tissue involvement were also visible (Figure 3a). The tumor invasion occurred in the connective tissue around the vessel and nerve bundles like the outer membranes. However, desmoid tumors around the vessels could not penetrate the vessel wall to form the tumor embolus (Figure 3b), whereas thicker nerve fibers were protected by their outer membrane. Perineural space was defined as the gap between the epineurium and the surrounding connective tissue. Desmoid tumors invaded into the connective tissue around the nerve bundles, perineural space, and even perineurium, but not into the nerve fibers (Figure 3c). Desmoid tumors with bone involvement penetrated the periosteum and cortical bone and invaded into the bone medullary cavity along the bone trabecula (Figure 3d).

### Immunohistochemical examination

**Figure 4:**
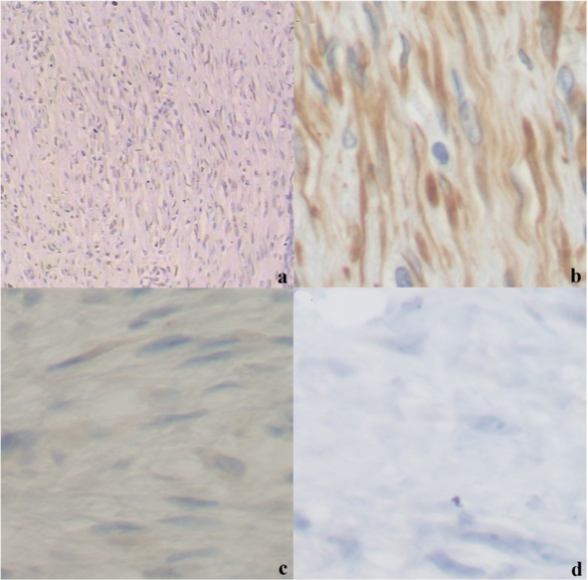
**Immunological features of desmoid tumors in the middle section of the left thigh of a 35-year-old woman with femur involvement (a–d). (a)** β-catenin staining of desmoid tumors (EnVision × 200). **(b)** Vimentin staining of desmoid tumors (EnVision × 200). **(c)** Desmin staining of desmoid tumors (EnVision × 200). **(d)** Ki-67 staining of desmoid tumors (EnVision × 200).

Generally speaking, β-catenin staining, among all the conventional stain methods for mesenchymal tumors, has proven to be the most effective stain method for the diagnosis of the desmoid tumors (data no shown). SMA staining, β-catenin staining, and Vimentin staining were positive in most lesions, whereas Ki-67 staining caused a low positive ratio, and Desmin staining was negative. Unexpectedly, β-catenin staining was rather specific (Figure 4a–d).

### Postoperative follow-up

Twenty two of the 56 patients with desmoid tumor showed postoperative recurrent symptom, whereas the other 34 were free of tumors. Thus, the recurrence rate was 39.3%. Primary treatment group had a recurrence rate of 30.7%, likewise which of the recurrent group was 46.7%. The relapse period ranged from 5 to 23 months (median 17.3 months) in the primary treatment group, whereas that of the recurrent group was from 3 to 26 months (median 14.8 months).

Univariate analysis showed that gender, patient age, primary treatment or recurrence, bone invasion, tumor site, tumor size, pharmacotherapy, and radiotherapy had no statistical significance on the recurrence (Table 3). The invasion of the major vessels and the quality of the surgical margins were statistically significant (*P* < 0.05, *X*^2^ = 6.766, 9.008, respectively). According to the logistic regression analysis, both aforementioned factors were independent variables for the recurrence [major vessels and nerves invasion *P* < 0.05, OR = 11.428, 95% CI (1.936, 67.459) (Tables 3 and 4); quality of the surgical margin *P* < 0.05, OR = 13.904, 95% CI (2.687, 71.951)]. Negative group had a lower recurrence rate (23.5%) than the positive one (63.6%). The group, free of major vessels and nerves, exhibited a lower recurrence rate (18.2%) than that with the involvement (52.9%).

**Table 3:** **Single**-**factor univariate analysis for the recurrence of desmoid tumors**

**Factors**	**Number of recurrence free**	**Number of recurrence**	***P*** **value**
	**(34 cases)**	**(22 cases)**	
Gender			
Male	12	8	0.935
Female	22	14	
Patient age			
< 30	18	8	0.224
≥ 30	16	14	
Clinical condition			
Primary tumor	18	8	0.224
Recurrence	16	14	
Tumor site			
Limb	26	14	0.299
Trunk	8	8	
Tumor size			
≤ 190 cm^3^	27	16	0.747
> 190 cm^3^	7	6	
Major vessels and nerves invasion			
No	18	4	0.020
Yes	16	18	
Bone invasion			
No	23	16	0.686
Yes	11	6	
Margin quality			
R0	26	8	0.007
R1/2	8	14	
Radiotherapy			
No	24	18	0.343
Yes	10	4	
Medication			
No	26	17	0.945
Yes	8	5

**Table 4:** **Logistic regression analysis of the recurrence rate of desmoid tumors**

**Factors**	**OR value**	***P*** **value**	**95% CI**
Major vessel and nerve invasion	11.428	0.007	1.936, 67.459
Margin quality	13.904	0.002	2.687, 71.951

## Discussion

Pathologically, it is easy to make a definite diagnosis of the desmoid tumors based on their morphological features and the immunohistochemical analyses of β-catenin. Budding-like lesions are visible on the juncture of desmoid tumors and muscles, which invade muscles and around adipose tissue aggressively along muscle bundles with the formation of small free focal lesions.

However, it is very hard to find such macroscopic lesions. The positive surgical margins may recur only if the resection is on the lesions themselves. Furthermore, tendon transposition and musculocutaneous flap transfer will be applied for partial reconstruction if the wide resection on the muscles causes the limb disturbance. In the case of desmoid tumors with deltoid and triceps muscle of arm involvement, Pruzansky et al. [8] removed a large section along the entire deltoid and three quarters of the triceps. The latissimus dorsi musculocutaneous flap was then applied to restore soft tissue and muscle function. Gallucci et al. [9] reported a case of aggressive fibromatosis in the proximal third of the forearm treated by wide resection and reconstructive surgery with no recurrence during a 3-year follow-up.

When the desmoid tumors invade the anadesma, ligament, joint capsule, or scar tissue, there is no obvious macroscopic boundary between the tumor cells and the normal tissue cells. Even under the microscope, it is hard to differentiate the tumor cells from the tendon ones if only based on the morphological observation. Therefore, during the surgery, a wider resection would be made with caution to avoid the residual disease. In terms of the recurrence patients, it is necessary to make a full resection as much as possible to remove the previous surgical scar. Artificial reconstructive ligament was not applied to restore the limb function and the joint stability until partial ligament or joint capsule was removed. In our study, the connective tissue around the larger vessel-nerve bundles was infected by the desmoid tumors, whereas the outer membranes of vessels and nerves were also invaded with the preclusion of vessel walls causing no tumor embolus.

The biological behavior of desmoid tumors is quite different from that of the other malignant tumors which devastate the vessels to form tumor embolus resulting in vascular metastasis. The reasons why desmoid tumors do not metastasize are also based on their biological behavioral characteristics. The thicker nerve fibers circumscribed by the outer membranes formed the perineural space, between which the around connective tissue locates. The tumors invade the around connective tissue of the nerve bundles, then the perineural space, and even the nerve bundle membrane. We also found that the small vessel and nerve branches were usually encapsulated by the tumors, which caused the outer membrane invasion of the vessel-nerve bundle. The tumors recur following the residue preservation. On the contrary, the surrounding connective tissues of the larger vessel-nerve bundles were abundant with the function of “protection.” If the tumors partially invade major vessels and nerves, the macroscopic tumor foci need to be removed and the vessels and nerves will be peeled off as much as possible (e.g. epineurium resection) to preserve the limb function. On the other hand, if the tissues receive a complete invasion, full resection of the tumors and the surrounding structures has to be conducted to decrease the recurrence rate. It may, however, be difficult for the functional reconstruction. Ferraresi et al. [10] reported a rare case that the radial nerve selectively invaded by the desmoid tumors was removed followed by graft repair with normal postoperative function and no recurrence during the 6-year follow-up. Desmoid tumors destroy the periosteum on the juncture of tumors and bones and then invade bone tissues along the bone trabecula with aggressive growth in the medullary cavity. Thus, partial bones will be removed, and the invasion of the medullary cavity needs to be examined attentively when the tumors and the bone tissues are close.

Time to relapse ranged from 8 to 23 months (median 17.3 months) in the primary treatment group, whereas that of the recurrence group was from 3 to 26 months (median 14.8 months). The recurrent patients presented at year 2 except for one relapsed after 26 months. Different from the malignant tumors such as osteosarcoma and chondrosarcoma, the relatively slow growth makes the patients with desmoid tumors less sensitive to radiotherapy or chemotherapy with late outcomes. As a result, the treatment and the postoperative follow-up should last at least 2 years.

The quality of surgical margins and the invasion of major vessels and nerves are the two independent risk factors causing the recurrence based on our statistical analysis of the clinical documents. The quality of surgical margins has also been regarded as the key factor affecting the postoperative outcomes. Nuyttens et al. [1] demonstrated that the local control rate of the negative-margin patient was higher than that of the positive with the preclusion of adjuvant radiotherapy. Pignatii et al. [3] found that the negative margins, rather than radiotherapy, gender, patient age, and tumor site, was the only factor to increase the local control rate. Mendenhall et al. [11] reviewed 10-year postoperative patients with surgery only and found that the local control rate of the negative margins (73%) was significantly different from that of the positive (46%). Thus, it is of critical importance in achieving the wide resection margins to control the desmoid recurrence rate.

Noticeably, the number of the chemotherapy cycle for desmoid tumors have not reached consensus, even though the newly developed adjuvant chemotherapy and radiotherapy have been applied extensively during the tumor treatment. Baliski et al. [12] reported a retrospective study on 13 desmoid tumor patients that 30 mg/day Adriamycin was infused preoperatively for 3 days, and then radiotherapy was used at a dose of 3000 cCy; surgery was applied 4–6 weeks after. Only two patients had a relapse during the average follow-up period of 71 months. The desmoid tumor control rate was as high as 85%, which was significantly higher than that of previous reports. Hence, the combined treatment strategy of preoperative chemotherapy and radiotherapy and surgery would be better than the traditional treatment, in other words, one of each aforementioned treatment [1].

Our study showed that the patients with desmoid tumor with vessel and nerve involvement had a comparatively high recurrence rate. One reason could be the vessels and nerves were preserved with their benign feature to avoid severe limb disturbance, which might leave the residual lesions in the vessel-nerve bundle surroundings or even inside causing local recurrence. Therefore, partial vessels and nerves need to be removed if necessary followed by local reconstruction to achieve the negative surgical margins.

## Conclusions

Our data showed that either the positive surgical margin or the tumors invading major vessels and nerves were able to cause the recurrence. Our suggestions for the therapeutic treatments are as follows: 1) simple soft tissue tumors need wide resection; 2) tendon transposition and musculocutaneous flap transfer will be conducted for partial reconstruction resulting from the soft tissue deficiency after wide resection; 3) if the tumors invade major vessels and nerves, the outer membrane resection for separation will be carried out to avoid severe disturbances; 4) when the tumors circumscribe vessels and nerves, full resection should be applied followed by partial reconstruction to cease the high recurrence rate; 5) if the tumors invade bone, lesion curettage, tumor segmental resection, inaction with bone replantation, or prosthetic replacement will be applied depending on the condition. Noticeably, the lesions in the medullary cavity should also be examined with caution. Briefly, radiotherapy, pharmacotherapy and other treatments may be used to control the local recurrence rate if the positive surgical margin or definitive surgery is not satisfying based on their biological behavioral features.

## Consent

Written informed consent was obtained from the patient for the publication of this report and any accompanying images.

## Abbreviations

NSAIDs: Nonsteroidal anti-inflammatory drugs
HE: Hematoxylin-eosin staining
TBS: Tris-buffered saline
MRI: Magnetic resonance imaging
CT: Computed tomography
SMA: Smooth muscle actin
